# Efficacy and safety of ^99m^Tc-MIBI SPECT-guided botulinum toxin injection in parkinsonian postural abnormality

**DOI:** 10.1186/s12883-026-05072-4

**Published:** 2026-06-17

**Authors:** Ronghua Hong, Zhuang Wu, Sha Zhu, Lizhen Pan, Yougui Pan, Xiaolong Zhang, Shuzhen Chen, Qiang Guan, Lingjing Jin

**Affiliations:** 1 Department of Neurology and Neurological Rehabilitation, Shanghai Disabled Persons’ Federation Key Laboratory of Intelligent Rehabilitation Assistive Devices and Technologies, Yangzhi Rehabilitation Hospital (Shanghai Sunshine Rehabilitation Center), School of Medicine, Tongji University, 2209 Guangxing Road, Shanghai, 201619 China; 2 Neurotoxin Research Center, Key Laboratory of Spine and Spinal Cord Injury Repair and Regeneration of Ministry of Education, Department of Neurology, Tongji Hospital, School of Medicine, Tongji University, 389 Xincun Road, Shanghai, 200065 China; 3 Department of Nuclear Medicine, Tongji Hospital, School of Medicine, Tongji University, Shanghai, China; 4 Collaborative Innovation Center for Brain Science (Sponsored by Shanghai Blue Cross Brain Hospital Co., Ltd. and Shanghai Tongji University Education Development Foundation), Tongji University, Shanghai, China

**Keywords:** Parkinson’s disease, Postural abnormalities, Botulinum toxin, ^99m^Tc-MIBI SPECT

## Abstract

**Background:**

Technetium-sestamibi single-photon emission computed tomography (^99m^Tc-MIBI SPECT) guidance has been shown to significantly improve the efficacy of botulinum toxin (BTX) injection in dystonic postural abnormalities. But its effect in parkinsonian postural abnormalities remains unknown.

**Methods:**

Eligible PD patients with postural abnormalities all underwent ^99m^Tc-MIBI SPECT muscle metabolic imaging and baseline evaluation followed by SPECT-guided BTX injection. The primary efficacy outcome was the improvement in resting forward and lateral trunk flexion angle (RTF and RTL) at 1 month. Secondary efficacy outcomes included subjective efficacy, pain VAS score and PDQ-39 score. Adverse events (AEs) were also assessed.

**Results:**

A total of 29 PD patients (14 males, 15 females) were analyzed with a mean age of 68.5 ± 8.9 years. The RTF increased (37.71 ± 16.82° vs. 40.17 ± 17.03°, Estimated Mean Difference = -2.46, 95% CI [-5.97, 1.06], *p* = 0.027) significantly while the RTL decreased (9.61 ± 8.78° vs. 8.28 ± 7.62°, Estimated Mean Difference = 1.34, 95% CI [-0.99, 3.67], *p* = 0.208) slightly at 1 month compared to baseline, though the difference was not significant. At 1 month, 4 patients (13.8%) experienced an improvement of more than 5° in RTF, 13 patients (44.8%) reported minimal to much subjective improvement and 10 patients (34.5%) saw an improvement of more than 1 point in the pain VAS score. Eleven AEs occurred, with no serious adverse events (SAEs). Related AEs mainly included transient worsening of postural abnormalities and low-back pain, which typically alleviated within 2 weeks to 2 months.

**Conclusions:**

The ^99m^Tc-MIBI SPECT-guided BTX treatment for parkinsonian postural abnormalities demonstrated acceptable safety but was not as effective as expected. Future studies should focus on identifying potential candidates who shall benefit from this method.

**Clinical trials registration:**

the Chinese Clinical Trial Registry, number ChiCTR2000039775, Nov. 8th, 2020.

## Introduction

Postural abnormalities, including sagittal abnormalities: camptocormia and anterocollis; coronal abnormalities: Pisa syndrome and scoliosis, are common disabling motor symptoms in patients with Parkinson’s disease (PD) and atypical parkinsonism [1, 2]. They manifest insidiously, progressing over time, often resulting in severe low back pain, worsening balance dysfunction and walking difficulties, which significantly impact the patients’ quality of life [3–5]. The pathogenesis is unclear, and axial dystonia is one of the possible hypotheses [6]. Conventional treatments primarily include drug therapy, surgical intervention, and rehabilitation therapy. However, the effectiveness of most of these methods remains unclear or limited [7–12]. Intervention of postural abnormalities in PD presents currently an urgent unmet need.

Different postural abnormalities result from the abnormal contraction of different muscle groups responsible for spasticity. Upper camptocormia, for instance, is likely due to excessive activity of the external oblique abdominal muscles, while lower camptocormia may result from overactivity of ventral hip muscles. Pisa syndrome, on the other hand, may be linked to hyperactivity of paraspinal muscles ipsilateral to the trunk leaning side [13, 14]. Botulinum toxin (BTX) injection has emerged as a potential treatment for these postural abnormalities, but existing studies are bothered by small sample sizes and inconsistent efficacy [15–17]. For instance, Azher et al. [18] treated 6 PD patients with camptocormia using botulinum toxin type A (BTX-A) in the rectus abdominis or/and paravertebral muscles, with only 3 patients showing moderate improvement. Coelln et al. [19] injected BTX-A into the iliopsoas muscle in 4 PD patients with camptocormia, yielding no benefits. Some studies, however, have reported relatively positive outcomes. A prospective open study demonstrated that bilateral external oblique muscle injection with BTX improved symptoms in 6 PD patients with camptocormia [20]. Artusi et al. supported the potential use of BTX injections in axial muscles to treat lateral trunk flexion in PD and suggested early intervention to prevent further deterioration [17]. The lack of accurate screening methods for identifying target muscles responsible for abnormal posture may be a crucial factor affecting efficacy.

Single-photon emission computed tomography (SPECT) muscle metabolic imaging, utilizing technetium-sestamibi (^99m^Tc MIBI) as the contrast agent, can reflect the blood flow perfusion and mitochondrial function in muscle cells, thereby displaying the abnormally contracted muscles. The ^99m^Tc-MIBI accumulation in skeletal muscle is proportional to blood flow perfusion and mitochondrial function. The tracer passively diffuses into cells and selectively binds to mitochondria, reflecting metabolic activity. During dystonia or hyperactivity, skeletal muscles exhibit increased contractility, leading to elevated regional blood flow and enhanced mitochondrial metabolism to meet energy demands. This metabolic upregulation results in increased ^99m^Tc-MIBI retention, enabling SPECT to identify regions of pathologically active muscle [21–24]. This method excels at detecting deep target muscles over traditional superficial electromyography [25]. Our previous work has demonstrated that ^99m^Tc-MIBI SPECT is a valuable method for screening target muscles in dystonic postural abnormalities, significantly improving the efficacy of BTX injection in patients with cervical dystonia [26, 27]. In this context, we aimed to explore the efficacy and safety of ^99m^Tc-MIBI SPECT-guided BTX injection in parkinsonian postural abnormalities at our center.

## Methods

### Study design

This is a prospective, single-arm, open-label, single-center study to investigate the efficacy and safety of ^99m^Tc-MIBI SPECT-guided BTX injection in PD patients with postural abnormalities at the Neurology Clinic of Tongji Hospital Affiliated to Tongji University. All participants underwent ^99m^Tc-MIBI SPECT muscle metabolic imaging and baseline evaluation. After that, they were treated with SPECT-guided BTX injection. Follow-up assessments were conducted at 2 weeks (T0), 1 month (T1) and 3 months (T2) after BTX treatment. The study was overseen by an independent data and safety monitoring board.

### Study participants

We included patients meeting the following inclusion criteria: (1) diagnosed of idiopathic PD according to Movement Disorder Society criteria [28]; (2) complicated with postural abnormalities defined as a reversible flexion of the trunk in the sagittal or coronal plane evaluated by a movement disorder expert; (3) can stand and walk without support for 2 min; (4) able to respond to instructions.

Main exclusion criteria were (1) with deformities, injuries or other diseases that may affect posture; (2) with medical history of mental disorders or schizophrenia that may affect the data collection.

### Sample size calculation

According to previous studies, the efficacy rate of botulinum toxin injection was about 40%, while the supposed efficacy rate in this study is 75% [29, 30]. Assuming a one-sided test level α = 0.025 (two-sided test level α = 0.05), a power (1-β) = 0.80, and a dropout rate of 10%, the estimated sample size is 30.

### Clinical evaluation

Clinical and demographic information, including age, body mass index (BMI), sex, levodopa equivalent daily dose (LEDD), and the duration of the disease were collected. The Hoehn & Yahr Stage (H-Y stage) was used to assess the disease severity of the patients [31]. The Movement Disorder Society-Sponsored Revision of the Unified Parkinson’s Disease Rating Scale part III (MDS-UPDRS III) was applied to assess the motor symptom [32]. The severity of pain is evaluated using the Visual Analogue Scale (VAS) [33]. Parkinson’s Disease Questionnaire-39 (PDQ-39) was applied for the quality of life [34]. The Clinical Global Impression of Change (CGI) scale was used by the clinician to record the patient’s assessment of the degree of improvement or worsening of postural abnormalities since baseline. The scale ranges from 3 to − 3, with a score of 3 defined as very much improved; 2, much improved; 1, minimally improved; 0, no change; −1, minimally worse; −2, much worse; and − 3, very much worse [35].

The primary efficacy outcome was the improvement in the resting forward and lateral trunk flexion angle (RTF and RTL) at 1 month. Secondary efficacy outcomes included CGI, pain VAS score and PDQ-39 score. Adverse events (AEs) were also assessed.

### Posture assessment

The posture features of all participants were collected with a motion analysis system which consisted of an Azure Kinect depth camera (depth camera 1024 × 1024 pixels @30fps, RGB 3840 × 2160 pixels @30fps, 7-microphone linear phased array, Microsoft), a guide screen, an independent computer, a frontal RGB camera (MCD-400 W plane, Ming Chuangda) and a lateral RGB camera (MCD-400 W plane, Ming Chuangda). The IFLYTEK Suzhou Research Institute has developed and provided a computer program that can acquire gait and posture motion characteristics of participants [36, 37].

All participants were asked to stand directly in front of the Kinect camera (at a distance of 2 m), after which we asked them to turn right at 90°. During this process, the motion analysis system was able to automatically collect the resting postural features of the patients. We collected the following two posture features of patients: (1) lateral trunk flexion angle, the angle between the connecting line of the 7th cervical spinous process (C7) and the 5th lumbar spinous process (L5) on the coronal plane and the vertical line of the ground (VL); (2) forward trunk flexion angle, the angle between the connecting line of C7 and L5 and the connecting line of lateral malleolus and L5 on the sagittal plane [36, 38].

All participants underwent anteroposterior and lateral whole-spine X-ray to rule out spinal fractures, and to determine the detailed pattern of postural abnormalities.

### ^99m^Tc-MIBI SPECT-guided botulinum toxin injection

Before the BTX treatment, ^99m^Tc-MIBI SPECT from the neck to the buttock was carried out according to the routine procedure reported by previous literature [24]. A muscle was defined as the target muscle when meeting the following two criteria: (1) the contraction of this muscle contributed to the abnormal posture from a pure mechanical perspective; (2) the muscle had higher radioactive uptake on ^99m^Tc-MIBI SPECT, compared to the same muscle on the other side judged by two experienced neurologists. Specifically, when criteria (1) and (2) could not be met at the same time, criterion (1) was followed first. The patient was then treated at the beginning (T0) by injection of 200–300 Units of Onabotulinum toxin-A, diluted in 4 mL of saline under the portable EMG (Haishen Medical, China) guidance, in the levator scapulae, sternocleidomastoid muscle, pectoralis major muscle, erector spinal muscle, external oblique muscle, internal oblique muscle, rectus abdominis, transversus abdominis, psoas quadratus muscle, psoas major muscle, iliac muscle, biceps femoris muscle, rectus femoris, semitendinosus or semimembranous muscle dependent on the ^99m^Tc-MIBI SPECT findings. Based on literature recommendations, the dose per muscle ranged from 25 to 100 units. To guarantee adequate dosage allocation, we limited the number of muscles injected per patient to a maximum of six during each session. The specific dosage for each muscle was determined by three criteria: anatomical size, functional importance, and spasticity severity. Consequently, larger or more severely affected muscles received higher doses within the recommended range [39].

### Statistical analysis

Data were analyzed by using the statistical package SPSS v.25.0. Measurement data are given as the mean (standard deviation), and qualitative data as counts of subjects and percentages. The statistical significance was set at *P* < 0.05. The Kolmogorov–Smirnov test was initially applied to check the normal distribution of measurement data. Clinical measurements and posture features obtained at each follow-up visit were compared using mixed linear models, with time as a fixed factor. To control for family-wise error rate (FWER) and false discovery rate (FDR) across multiple outcomes and repeated time points, adjustments for multiple comparisons were performed using the Holm-Bonferroni step-down procedure for time-dependent comparisons and the Benjamini-Hochberg (BH) procedure for multiple outcome measures. Given the non-normal distribution of between-group differences, pairwise comparisons were assessed using the Wilcoxon Signed Rank Test, with adjusted P-values calculated post-hoc based on the BH and Holm-Bonferroni corrections. At 3 months after treatment, seven patients were lost to follow-up; missing data were filled using the last observation carried forward (LOCF) method. Considering the progressive nature of parkinsonian postural abnormalities and the absence of effective treatments, implying a worsening trajectory. LOCF provides a conservative estimate, avoiding overestimation of treatment effects.

## Results

### Demographic and baseline clinical characteristics

**Fig. 1:**
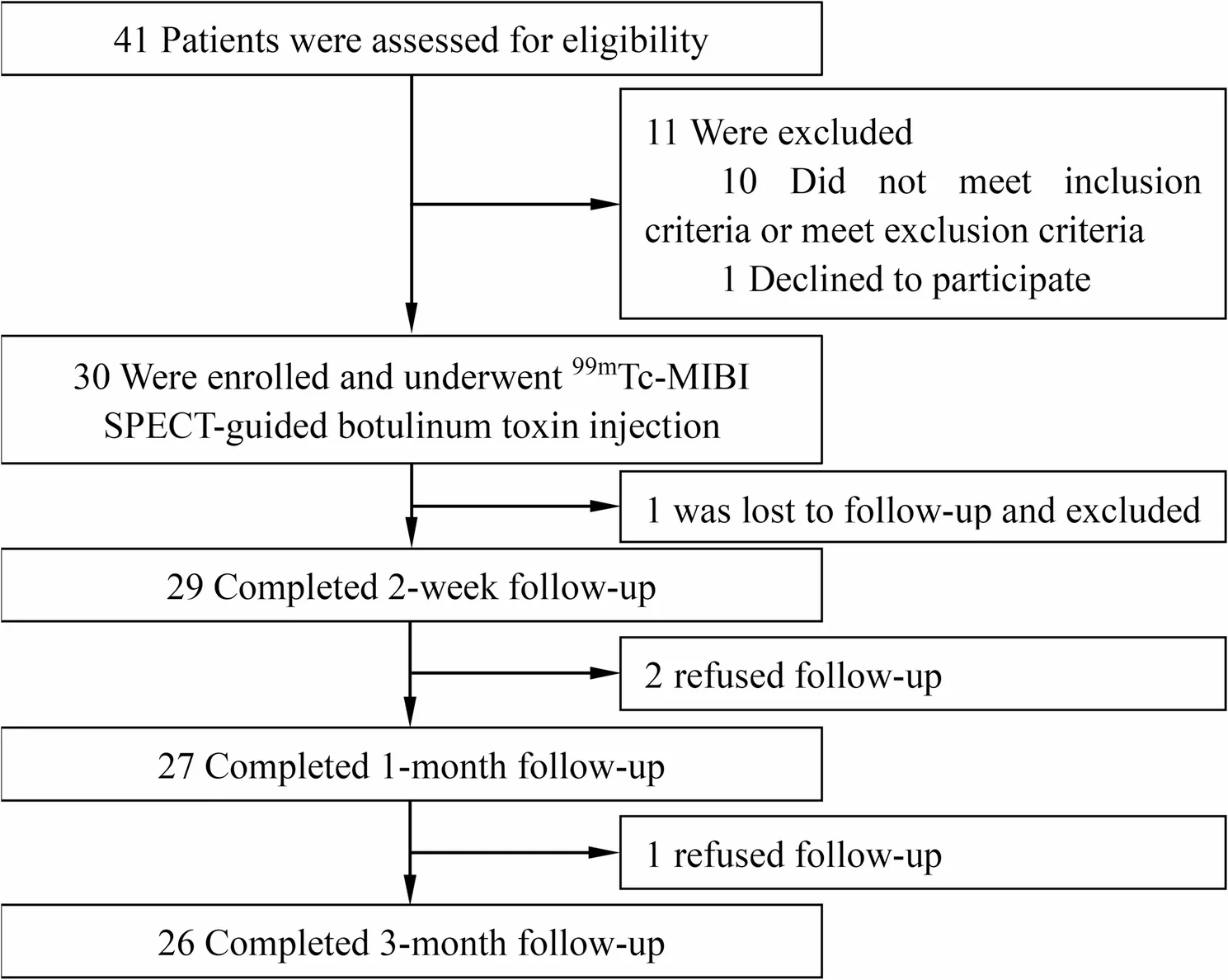
Study flowchart

A total of 41 patients were initially screened for eligibility at the Neurology Clinic of Tongji Hospital Affiliated to Tongji University. Thirty participants were enrolled in the study from Dec. 17th, 2020 to Apr. 26th, 2023 (Fig. 1). Eventually, 29 participants (14 males, 15 females) were included in the analysis. The analyzed participants had a mean age of 68.5 ± 8.9 years and a mean disease duration of 9.9 ± 5.9 years. The mean H-Y stage and MDS-UPDRS part Ⅲ score of the patients was 2.5 ± 0.8 and 42.3 ± 15.2, respectively. The Visual Analogue Scale (VAS) score for back pain was 5.3 ± 3.1. Detailed demographic and clinical data before treatment (T0) are presented in Table 1.

**Table 1:** Demographic and clinical characteristics of the participants at baseline (T0)

*N*	Total	Camptocormia	Pisa syndrome
	29	17	12
Age, years	68.5 ± 8.9	68.6 ± 9.8	68.3 ± 7.8
Female, n(%)	15(51.7)	7(41.2)	8(66.7)
BMI, kg/m2	24.6 ± 4.3	24.5 ± 4.3	24.7 ± 4.4
Disease duration, years	9.9 ± 5.9	9.8 ± 5.6	10.0 ± 6.2
Levodopa equivalent daily dose, mg	536.2 ± 261.0	533.8 ± 261.4	540.6 ± 272.1
H-Y stage	2.5 ± 0.8	2.5 ± 0.8	2.6 ± 0.8
MDS-UPDRS part Ⅲ score	42.3 ± 15.2	42.1 ± 15.5	42.5 ± 15.5
VAS score	5.3 ± 3.1	5.3 ± 3.1	5.3 ± 3.2
PDQ-39 score	55.5 ± 31.3	55.2 ± 31.5	55.9 ± 32.3

The data shown are mean ± Standard Deviation or count (% of total)

*Abbreviations*: *N* Number, *BMI* Body mass index, *H-Y* Hoehn & Yahr, *MDS-UPDRS* The Movement Disorder Society-Sponsored Revision of the Unified Parkinson’s Disease Rating Scale, *VAS* Visual analogue scale, *PDQ-39* Parkinson’s Disease Questionnaire-39

### Efficacy outcomes

**Fig. 2:**
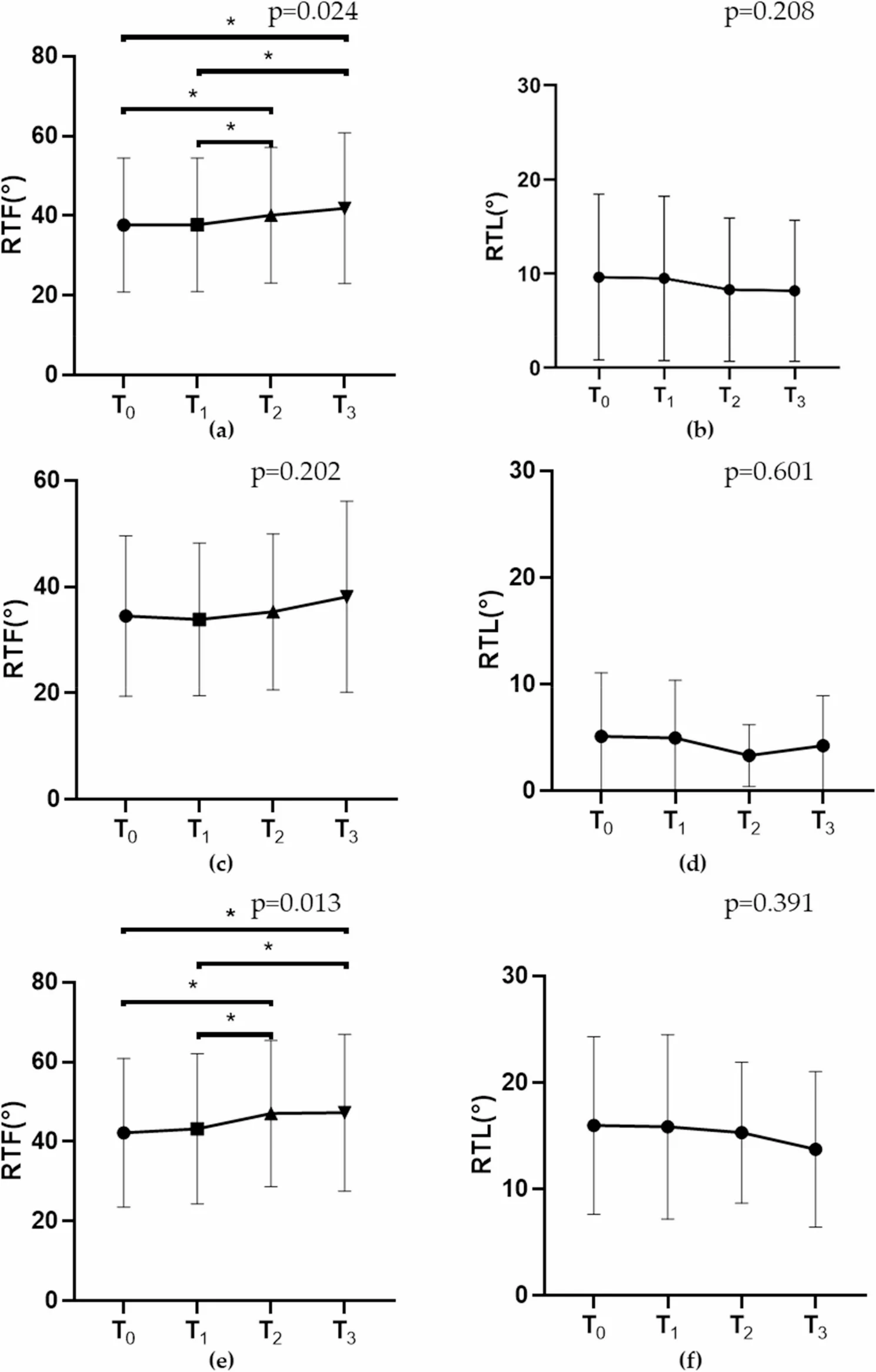
Changes in postural features after BTX treatment. **a** Changes in RTF in all patients; (**b**) Changes in RTL in all patients; (**c**) Changes in RTF in camptocormia group; (**d**) Changes in RTL in camptocormia group; (**e**) Changes in RTF in Pisa syndrome group; (**f**) Changes in RTL in Pisa syndrome group. *Abbreviations*: *BTX* Botulinum toxin, *RTF* Forward trunk flexion angle at rest state, *RTL* Lateral trunk flexion angle at rest state

For the first efficacy outcome, we found that the RTF increased (37.71 ± 16.82° vs. 40.17 ± 17.03°, Estimated Mean Difference = -2.46, 95% CI [-5.97, 1.06], *p* = 0.027) significantly while the RTL decreased (9.61 ± 8.78° vs. 8.28 ± 7.62°, Estimated Mean Difference = 1.34, 95% CI [-0.99, 3.67], *p* = 0.208) slightly at 1 month compared to baseline, though the difference was not significant (Fig. 2a, b). We have also conducted separate analyses for the camptocormia and Pisa syndrome groups. While minor differences in statistical significance were observed between the two subgroups, the overall statistical trends remained consistent (Fig. 2c-f).

**Fig. 3:**
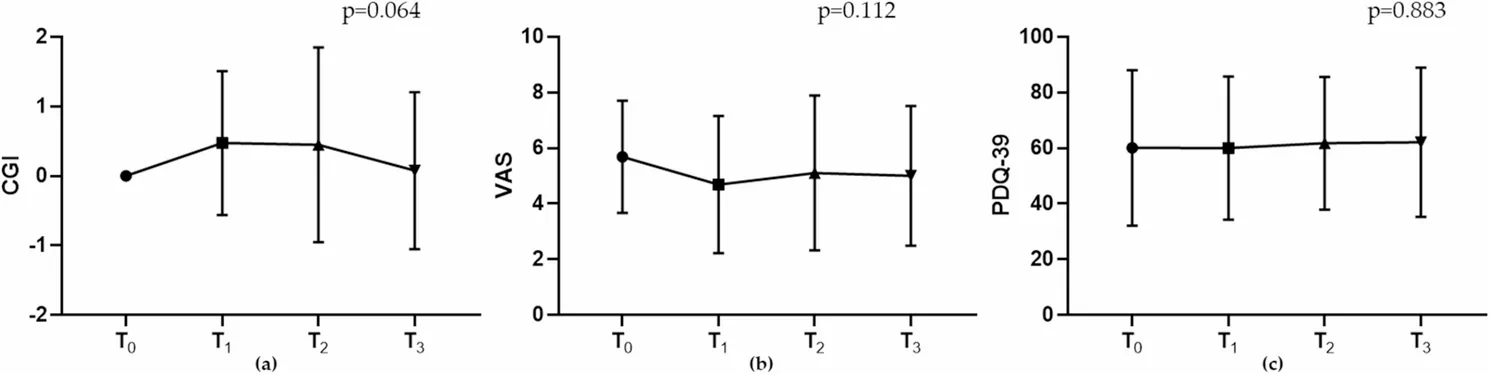
Changes in clinical ratings after BTX treatment. **a** Changes in CGI; (**b**) Changes in VAS score; (**c**) Changes in PDQ-39 score. *Abbreviations*: *CGI* Clinical Global Impression of Change, *VAS* Visual analogue scale, *PDQ-39* Parkinson’s Disease Questionnaire-39

For the secondary efficacy outcomes, although there was a mild trend of improvement in CGI and VAS score at 2 weeks, there was no statistical difference among the four time points in terms of CGI (*p* = 0.064), VAS score (*p* = 0.112), and PDQ-39 score (*p* = 0.883) (Fig. 3).

At the individual level, 4 patients (13.8%) experienced an improvement of more than 5° in RTF, 13 patients (44.8%) reported minimal to much subjective improvement and 10 patients (34.5%) saw an improvement of more than 1 point in the pain VAS score at 1 month (Table 2).

**Table 2:** Summary of participants benefiting from botulinum toxin treatment

	T0	T1	T2	T3
CGI improved by more than 1 point	NA	14(48.3)	13(44.8)	7(24.1)
VAS improved by more than 1 point	NA	13(44.8)	10(34.5)	10(34.5)
RTF improved by more than 5°	NA	3(10.3)	4(13.8)	3(10.3)
RTL improved by more than 3°	NA	2(6.8)	6(20.7)	8(27.6)

The data shown are count (% of total)

*Abbreviations*: *CGI* Clinical Global Impression of Change, *VAS* Visual analogue scale, *RTF* Forward trunk flexion angle at rest state, *RTL* Lateral trunk flexion angle at rest state, *NA* Not applicable

### Safety outcomes

Regarding safety outcomes, a total of 11 adverse events (AEs) were recorded, with no instances of serious adverse events (SAEs) identified. The predominant AEs were transient exacerbation of postural abnormalities and low-back pain, typically resolving within two weeks to two months without necessitating intervention. It is noteworthy that three patients deviated from the prescribed injection plan outlined in criterion (1). Specifically, they received injections in the erector spinal muscle (according to the findings of SPECT) contralateral to the leaning side, a deviation that lacked mechanical rationale. Importantly, all three patients experienced a transient worsening of postural abnormalities.

### Preliminary analysis of the difference between the favorable group and unfavorable group

The patients were further divided into two group based on the CGI at 1 month after BTX injection. The favorable group (*n* = 13) was defined as the CGI ≥ 1 while unfavorable group (*n* = 16) was defined as the CGI<1. The favorable group had a marginally significantly younger mean age (66.38 ± 7.33 vs. 70.88 ± 4.83, *p* = 0.058) and a significantly younger onset age than the unfavorable group (56.08 ± 6.97 vs. 61.37 ± 5.83, *p* = 0.034). The injection rate of the internal oblique muscle in the favorable group was marginally significantly higher than that in the unfavorable group (76.90% vs. 43.80%, *p* = 0.071), demonstrating that internal oblique muscle injection may serve as a favorable factor for treatment efficacy. Notably, as mentioned above, all three patients whose injection plan did not follow the mechanical rationale inevitably belonged to the unfavorable group. The preliminary analysis of the difference between the two groups were listed in Table 3.

**Table 3:** Preliminary analysis of the difference between the favorable group and unfavorable group

	Favorable group	Unfavorable group	*p* value
*N*	13	16	NA
Age, years	66.38 ± 7.33	70.88 ± 4.83	0.058
Onset age, years	56.08 ± 6.97	61.37 ± 5.83	0.034*
Female, n(%)	5 (38.50%)	10 (62.50%)	0.198
BMI, kg/m2	23.09 (2.52)	23.74 (3.40)	0.569
Disease duration, years	10.31 ± 3.22	9.69 ± 4.87	0.697
Levodopa equivalent daily dose, mg	565.38 ± 186.12	475.78 ± 197.61	0.223
H-Y stage	2.73 ± 0.93	2.59 ± 0.55	0.626
MDS-UPDRS part Ⅲ score	43.73 ± 20.61	41.63 ± 13.88	0.753
Levator scapulae IR, n(%)	3 (23.10)	0 (0.00)	0.157
Sternocleidomastoid muscle IR, n(%)	0 (0.00)	1 (6.30)	1.000
Pectoralis major muscle IR, n(%)	1 (7.70)	0 (0.00)	0.916
Erector spinal muscle IR, n(%)	5 (38.50)	4 (25.00)	0.707
External oblique muscle IR, n(%)	7 (53.80)	9 (56.30)	0.897
Internal oblique muscle IR, n(%)	10 (76.90)	7 (43.80)	0.071
Rectus abdominis IR IR, n(%)	9 (69.20)	9 (56.30)	0.740
Transversus abdominis IR, n(%)	1 (7.70)	1 (6.30)	1.000
Psoas quadratus muscle IR, n(%)	0 (0.00)	1 (6.30)	1.000
Psoas major muscle IR, n(%)	6 (46.20)	7 (43.80)	0.897
Iliac muscle IR, n(%)	1 (7.70)	5 (31.30)	0.273
Rectus femoris IR, n(%)	0 (0.00)	1 (6.30)	1.000
Biceps femoris muscle IR, n(%)	0 (0.00)	2 (12.50)	0.559
Semitendinosus muscle IR, n(%)	0 (0.00)	2 (12.50)	0.559
Semimembranous muscle IR, n(%)	0 (0.00)	2 (12.50)	0.559
Patients whose injection plan violated the mechanical rationale, n(%)	0 (0.00)	3 (18.75)	0.120

The data shown are mean ± Standard Deviation or count (% of total). *p<0.05, **p<0.01, ***p<0.001

*Abbreviations*: *N* Number, *NA* Not applicable, *BMI* Body mass index, *H-Y* Hoehn & Yahr, *MDS-UPDRS* Movement Disorder Society-Sponsored Revision of the Unified Parkinson’s Disease Rating Scale, *IR* Injection rate

## Discussion

To the best of our knowledge, this is the first study to explore the efficacy and safety of ^99m^Tc-MIBI SPECT-guided BTX treatment for parkinsonian postural abnormalities. Finding from this single-arm, open-label 12-week investigation indicate that ^99m^Tc-MIBI SPECT-guided BTX treatment for parkinsonian postural abnormalities exhibits acceptable safety but only demonstrates efficacy primarily in specific patients. Despite a lack of significant improvement in forward and lateral trunk flexion angles on a group level, a considerable number of patients derived benefits, particularly in terms of CGI improvement and relief from low back pain. These results underscore the necessity of discerning individuals who will benefit from this treatment, underscoring the importance of personalized treatment strategies.

The existing literature on BTX treatment for parkinsonian postural abnormalities is limited, predominantly comprising case reports or small-sample single-arm open-label studies with variable findings [17, 20, 40–43]. For example, Santamato et al. described a case of a 68-year-old man with PD combined with severe axial lateral trunk flexion who benefited from a combination of BTX injection and rehabilitation [40]. In another study, BTX-A was injected into ipsilateral iliocostalis muscles with 50U in each site (T10 and L3), and the patient reported pain release and functional improvement, but without significant improvement of lateral flexion [42]. Additionally, Artusi et al. enrolled 13 PD patients with lateral trunk flexion (LTF) > 5° [17], which received ultrasound- and electromyography-guided BTX injections every 4 months, and 7 matched PD patients with LTF as controls. Overall, BTX-treated patients obtained a not statistically significant improvement of LTF of 17 ± 41% (*p* = 0.237). In comparison, the seven untreated PD patients suffered a deterioration in LTF over 12 months by 36 ± 45% (*p* = 0.116), showing a significantly different trajectory of posture change (*p* = 0.026). A study evaluated the efficacy of BTX under MRI, US- and EMG-guided injections in 15 PS patients [29]. 13 patients finished follow-up, and 84.6% of them ameliorated at least 5 degrees, while pain improved in all patients [29]. In our study, there was a downward trend of RTL after BTX injection among different follow-up time points, though the difference was not statistically significant compared to the baseline. This suggested that ^99m^Tc-MIBI SPECT-guided BTX treatment in parkinsonian postural abnormalities showed efficacy at least for certain patients. The trajectory of lateral trunk flexion improvement varies among individuals, emphasizing the heterogeneous nature of parkinsonian postural abnormalities.

Concerning camptocormia, the efficacy of BTX is less promising, with conflicting reports. For instance, one study employing ultrasound-guided ventral BTX injection into the iliopsoas muscle of 4 PD patients with camptocormia did not yield substantial and enduring posture improvement. The authors concluded that injection of BTX into the iliopsoas did not appear to be a promising approach for the treatment of parkinsonism-associated camptocormia [19]. Conversely, Todo et al. enrolled 6 patients with PD and flexion of the thoracic spine. BTX-A (75 or 90 units, onabotulinum toxin A) were injected into each external oblique (EO) bilaterally under sonographic guidance. Two weeks after the injection, the mean camptocormia angle showed significant attenuation [median (interquartile range); 38° (23.5°) vs. 18° (21°), *p* = 0.028]. Subjective relief was present in cases 1–3 and 6, and absent in cases 4 and 5. In our study, ^99m^Tc-MIBI SPECT-guided BTX treatment did not significantly reduce the forward trunk flexion angle on average. Its impact on the progression of camptocormia remained unknown due to the lack of controls.

It is noteworthy that increased RTF typically indicates worsening camptocormia in Parkinsonian postural abnormalities. In our study, we observed a group-level increase in RTF, which may reflect natural disease progression or treatment-related effects in some patients. However, we also identified individuals who demonstrated decreased RTF with concurrent subjective improvements, suggesting heterogeneous responses to botulinum toxin injections. The group-level RTF increase may be driven by patients who experienced disease worsening or unintended muscle weakness from injections, as prior literature has reported mixed botulinum toxin effects on posture [17, 20, 40–43]. Conversely, subjective improvements in select cases may result from targeted muscle relaxation reducing abnormal postural tension, as supported by patient-reported outcomes and clinician assessments. The SPECT-guided approach aims to optimize anatomical targeting, potentially explaining beneficial responses in specific patients. This finding underscores the importance of personalized treatment strategies and multi-modal assessments in parkinsonian posture management.

The concerns regarding natural progression, placebo effects, and regression to the mean are indeed critical when interpreting outcomes from a single-arm, open-label study. First, regarding the natural progression of postural abnormalities, extensive literature including our 3-year follow-up of 151 PD patients indicates that Parkinsonian postural abnormalities are typically progressive and notoriously resistant to conventional dopaminergic therapy [3, 4, 44]. Therefore, the improvements observed in some of our patients over 3 months are highly unlikely to represent spontaneous remission or a benign natural course of the disease. Second, while we acknowledge the potential for placebo effects, the objective nature of our primary outcomes and the specific mechanism of BTX action help mitigate this concern. Finally, the possibility of regression to the mean is considered lower in this specific patient population. Patients with advanced PD and established postural abnormalities represent a stable, chronically impaired state rather than a fluctuating acute condition, making a significant spontaneous improvement toward a “true” mean less probable.

Importantly, in our study, three patients that received injections in the erector spinal muscle (according to the findings of SPECT) contralateral to the leaning side, all experienced a worsening of their condition without exception. Based on this finding, we did not recommended injection of muscles that did not make sense from a mechanical perspective even though the muscle’s radioactive uptake on ^99m^Tc-MIBI SPECT was high, which is probably compensatory for the trunk leaning [6, 14].

Despite its limitations, including a small sample size and the absence of a placebo comparator group, this study pioneers the application of ^99m^Tc-MIBI SPECT-guided BTX treatment in parkinsonian postural abnormalities. The objective measurement of trunk flexion degrees, along with the concurrent assessment of forward and lateral trunk flexion, contributes new insights to the existing evidence.

## Conclusions

In summary, ^99m^Tc-MIBI SPECT-guided botulinum toxin treatment for parkinsonian postural abnormalities demonstrates acceptable safety and appears effective only for specific patient groups. Future investigations should delve into the factors contributing to variation in BTX efficacy, aiming to craft more effective and safer individualized treatment.

## Data Availability

The datasets generated during the current study are available from the corresponding author on reasonable request.
